# Nanomaterials in Drug Delivery Applications

**DOI:** 10.3390/nano12203565

**Published:** 2022-10-12

**Authors:** André Ferreira Moreira

**Affiliations:** 1 CICS-UBI—Health Sciences Research Centre, University of Beira Interior, 6200-506 Covilhã, Portugal; afmoreira@fcsaude.ubi.pt; 2 CPIRN-UDI/IPG—Center of Potential and Innovation in Natural Resources, Research Unit for Inland Development, Polytechnic Institute of Guarda, Avenida Dr. Francisco de Sá Carneiro, No. 50, 6300-559 Guarda, Portugal

The “magic bullet” concept paved the way for nanomaterials’ development and innovation. Nanomaterials, with sizes ranging between 1 and 100 nm, are currently being applied in different medical applications, such as drug delivery, biosensors, biomarkers, and imaging. The nanoscale size of materials results in unique physicochemical (e.g., mechanical, chemical, magnetic, electrical, optical) and biological properties, and there is an abundance of articles showcasing the development, discovery, and characterization of different nanomaterials for various biomedical applications. Moreover, over the last 30 years, researchers have been exploring the development of nanomaterials with different structures (2D and 3D), organizations, and materials, such as carbon-based (nanotubes and graphene), silica oxides (porous silica and organosilica), metal oxides (gold and iron), nanocrystals, lipids (liposomes and solid lipids), polymers (micelles and drug-polymer conjugates), dendrimers, and quantum dots. 

This Special Issue will cover the application of nanoparticle-based drug delivery systems, from new synthesis methodologies to toxicological evaluation. The nano-sized drug delivery systems can enhance the drugs’ solubility, which is a result of their small particle size and large surface area, and blood half-life with minimal off-target effects or toxicity to healthy tissues. Crintea and colleagues reviewed the nanoparticles’ applicability to improve the efficacy of the vitamins and increase the patient’s compliance [1]. This revision compares the different types of nanoparticles that have been explored for vitamin D and K transportation, addressing the benefits and shortcomings of each approach. Additionally, the nanomaterials can also be engineered to cross the blood–brain barrier, enter the pulmonary system, penetrate the tumors, and transpose the tight junctions of skin endothelial cells. In this regard, Uchida and coworkers introduced the application of physical perturbations or nanocarriers to enhance transdermal drug delivery [2]. In this work, the authors discussed the role of the nanocarriers’ flexibility, shape, and size, as well as the ideal properties for a nanocarrier to effectively transpose the skin tissue. Moreover, the nanomaterials also protect their payload from premature degradation and result in more controlled drug clearance, which contributes to an increased accumulation at the target site. Huang et al. implemented a coaxial electrospraying method to form ultra-thin ethyl cellulose (EC) on a medicated composite core consisting of tamoxifen citrate (TAM) and EC [3]. The EC coating resulted in a sustained release of Tamoxifen, increasing the time to release 50% of the drug from 1.89 h to 12.79 h. Therefore, the utilization of nanomaterials provides new approaches for researchers to tailor pharmacokinetics and pharmacodynamics, which can also be an essential step for drug repurposing and the development of more effective therapeutics. Moreover, nanomaterials can also be engineered to combine the drug delivery capacity with other therapeutic approaches, such as photothermal therapy, as demonstrated by Fernandes and colleagues with the development of mesoporous silica-coated gold nanoclusters [4].

Despite all the enthusiasm behind the nano-sized drug delivery systems, their translation from the laboratory to the clinic has been challenging. This gap can partially be justified by the limited significance between animal and human studies, namely the physiological and pathological differences influencing the nanomaterials’ behavior in the body. Otherwise, the heterogeneity between the patients and the biological characteristics of the disease can also negatively impact the clinical success of nanomedicines. Rudi and coworkers further evaluated the effect of unmodified and functionalized *Spirulina platensis* biomass silver nanoparticles on rats during prolonged oral administration [5]. The authors observed that the biofunctionalized particles had higher accumulation in the brain and spleen, whereas the unmodified ones showed higher affinity for the liver and kidneys. Moreover, both silver nanoparticles provoked changes in the renal and hepatic parameters, which indicates probable toxicity issues.

However, it is worth noticing that the continuous development of more precise nanomaterials and approval of novel nanomedicines (e.g., COVID-19 BNT162b2 mRNA vaccine) will generate important data for improving bioavailability, introducing smart-release profiles (stimuli responsivity for on-demand drug release), minimizing unwanted toxicities and ultimately widening the nanomaterials application and improving patient outcome.

## Data Availability

Not applicable.
